# S10-3 Research on policy audits for promoting physical activity at the local level in Japan

**DOI:** 10.1093/eurpub/ckad133.050

**Published:** 2023-09-11

**Authors:** Noriko Takeda, Yukio Oida, Shigeru Inoue, Motohiko Miyachi

**Affiliations:** Kogakuin University; Chukyo University; Tokyo Medical University; Waseda University

## Abstract

**Purpose:**

Increasing levels of health-enhancing physical activity (HEPA) requires a comprehensive policy framework across all political levels. At the local level, Japan has a two-tier system of government: prefectures and municipalities. We developed specific tools to identify the strengths and weaknesses of the implementation of national HEPA policies by the different local governments.

**Methods:**

We started by developing and testing the Local Policy Audit Tool (L-PAT) in all 47 Japanese prefectures (Takeda et al. 2019). It comprises 11 items based on WHO’s HEPA Policy Audit Tool (PAT), covering key policy actions such as: the presence of a local action plan; its implementation; and cross-sectoral cooperation. The L-PAT was sent to key government departments (e.g. health, sports, urban planning and transport) in all prefectures. As a second step, we assessed the development and implementation of local action plans for HEPA promotion in municipalities using the City PAT (C-PAT), which employs six questions based on the HEPA PAT. We surveyed a random selection of 272 out of Japan’s 1,700+ municipalities, stratified by administrative division and population.

**Results:**

The results of the L-PAT indicate that health and sports are the most active departments in promoting HEPA policy at prefecture level. Some prefectures also reported HEPA actions by the urban planning and transport sectors, although these sectors are not covered by the national policy. A major result of the C-PAT survey was that the scale and contents of projects implemented differed depending on the size of the municipality. Both tools revealed that local governments have a low awareness of the “Japanese physical activity guidelines for health promotion 2013” in their policy planning and implementation.

**Conclusions:**

In 2023, both the Japanese physical activity guidelines and the government's most comprehensive health policy, “Healthy Japan 21”, will undergo major revisions. The next step after the L-PAT and C-PAT would be to revise the content of the C-PAT questionnaire to increase its usefulness; that is, a policy audit tool for continuously monitoring local HEPA policies will be created on this occasion, aimed at supporting future national planning processes for HEPA promotion.

